# 

**Published:** 1880-06

**Authors:** 


### INDEX TO VOLUME XL.

A.

PAGE.

Anæsthetics. two new, by J. C. Reeve, M.D ... 628
Anæsthetic, hydrobromic ether as an ... 429
Abscess, follicular, of the urethra, by Prof. F. N. Otis, M.D. 459
American medical association .. 444
Allen, Prof. J. Adams ... 192
Andrews, Edmund, M.D., Japanese surgery and medicine ... 87
Announcements for the months, 112, 224, 336, 448, 560, 672
Acoustic illusion, a curious ... 599
Alcohol as a disease, by S. F. Bennett, M.D ... 359
Anomalous origin of the descendents noni, by G. Frank Lydston, M.D ... 263
Adolphus, Philip, M.D., the mechanical adaptation of the obstetrical forcepts ... 152
Alumni association of Rush Medical College ... 437
Audiphone, the, and kindred instruments, by E. L. Holmes, M.D ... 363
Audiphone, the, by E. Fletcher Ingals, M.D ... 76

B.

Barlow, A., M.D., poison by chloras potassii ... 518
Bartlett, Jno., M.D., the mechanics of the obstetrical forceps. 225
Baldwin, A. E., M.D., report of a case of lithotomy ... 73
Belfield, W. T., M.D., Vienna letter ... 619
Bennett, S. F., M.D., alcohol as a disease ... 359
Belfield, W. T., M.D., expert testimony in the Hayden trial ... 145
Blood letting in the treatment of pneumonia, by T. F. Worrell, M.D ... 354

PAGE.

Bridge, Dr. Norman, Cook Co. Hospital staff ... 508
Books and Pamphlets received. 99, 191, 385, 527, 626
Book Notices and Reviews:
- Ophthalmic, out-patient practice, by Chas. Higgins, F.R.C.S ... 625
- Hearing, and how to keep it, by E. H. Burnett, M.D ... 624
- The student's guide to diseases of the eye, by E. Nettleship, F.R.C.S ... 622
- Diphtheria, its nature and treatment, by Morell Mackenzie, M.D ... 621
- The pathology and treatment of venereal diseases, by F. J. Bumstead, M.D ... 182
- A systematic treatise on the diagnosis and treatment of diseases, by Austin Flint, M.D ... 177

Boston letter ... 168, 521
Brower, D. R., M.D., a new surface thermometer ... 505
Book Notices and Reviews ... 275, 384
Buboes, the treatment of indodolent, by E. J. Doering, M.D. 369
Burns and scalds, the early treatment of, by E. F. Ingals, M.D.. 249
Bulkley, L. Duncan, M.D., on the use of water in the treatment of diseases of the skin ... 52
Buck, A. H., M.D., hygiene and public health, edited by ... 89

C.

Capsicum, hemorrhoids treated with ... 549
Cataract, congenital nuclear ... 525
Crandall, J. B., M.D., a case of triplets ... 507

PAGE.
Cæsarean operation, the prognosis of.................... 365
Changes, editorial.............. 101
Clevenger, S. V., M.D., chemistry and toxicology of mercury... 337
Chicago Medical College...... 439
Cinchona cure for intemperance, by Chas. Warrington Earle, M. D......................... 128
Criminals, experiments upon... 110
Concours at Rush Medical College.................117, 626
Croup and diphtheria, cases of tracheotomy for, by E. F. Ingals, M.D.................... 594
Convulsions, infantile.......... 533
Congenital nuclear cataract ... 525
Cook county hospital staff, Dr. Norman Bridge............. 508
Contamination of drinking water by filtration of organic matter through the soil, by Victor C. Vaughn, M.D....... 635
Correspondence, domestic, Philadelphia letter.............. 377
Conant, Jno., small pox, a suggestion.................... 274
Cyclopædia, American, of domestic medicine............. 98

D.

Davis, N. S., M.D., LL.D., an address upon the science and art of medicine............. 449
Day, E., M.D., a severe case of puerperal eclampsia at the eigthth month of pregnancy, recovery.................... 597
Dental hints for country practitioners, by G. Newkirk, M.D.. 63
Diphtheria and croup, cases of tracheotomy for, by E. F. Ingals, M.D.................... 594
Diseased meat and foul water considered as causes of disease, by H. M. Lyman, M.D........ 588
Diphtheria, new treatment of... 386
Dilatation of the left ventricle, was it? by K. J. Thomas, M.D. 374
Disease, alcoholism as a, by S. F. Bennett, M.D............... 359
Diphtheria, the treatment of, by Dr. Rudolph Seiffert....... 166
Drinking water, contaminated by filtration of organic matter through the soil, by V. C. Vaughan, M.D............. 635
Double monsters, by L. S. Pilcher, M.D................. 416

PAGE.
Doering, E. J., M.D., the treatment of indolent buboes..... 369

E.

Examinations of the Illinois state board of health.. .... 620
Earle, Chas. Warrington, M.D., the cinchona cure for intemperance................ 128
Expert testimony in the Hayden trial, by Wm. T. Belfield, M.D. 145
Editorials........................ 627
Epidemics, local sanitary conditions and.................. 534
Editorials, the abuse of medical chairities.................. 528
Editorials, code of ethics of Massachusetts state medical society.................... 387
College sociability.......... 391
Mrs. Astor's present to New York academy of medicine. 387
Editorials, medical charities... 281
Editorial....................... 100
Ergotine, treatment of prolapsus of the rectum by hypodermic injections of............ 590
Ergot, the value of, in the treatment of pneumonia, by Wm. Meacher, M.D............... 260
Eye, rheumatic diseases of the, value of salicylic acid in ; by F. C. Hotz, M.D............. 501

F.

Fenger, Chr., and E. W. Lee, select topics of modern surgery.... .................118 465
Fitch, W. H., M.D.; excision of spina bifida tumor ; cure.... 371
Foul water and diseased meats considered as causes of disease; by H. M. Lyman, M.D.. 588
Fourth year a, in the medical school; by J. C. White, M.D.. 547
Forceps, the mechanics of the Obstetrical; by Jno. Bartlett, M.D........................ 225
Forceps, the mechanical adaptation of the obstetric; by Philip Adolphus, M.D........ 152

G.

Genesis of ovarian cysts; diseases of blood vessels in relation to...................... 556
Gibney, V. P., M. D., perinephritis; 15 additional cases in children, making a total of 28 cases...................... 361

PAGE.
Godfrey, H. F., M.D., obstruction of bowel (probably intussusception) relieved by aspiration ........ 373
Gynæcology, the diagnosis and treatment of obstetric cases by external manipulation... 661
Gynæcology, displacements of the uterus; a new theory of their mechanism and treatment ........ 659
Gynæcology and obstetrics; treatment of, anti and retroversions and of anti and retroflections of the uterus ....... 555
Gynæcological society of Chicago ........ 509
Gynæcology in the seventeenth century, by Dr. James I. Tucker ........ 154

H.

Hemorrhoids treated with capsicum ........ 549
Hotz, F. C.. M.D., value of salicylic acid in rheumatic diseases of the eye ........ 501
Hospital reports, Aberdeen royal infirmary, naso pharyngeal polypi, ecrasement and osteo-plastic operation, rapid recovery ........ 413
Hospital reports, Ashton-Under-Lyne district infirmary... 408
Hospital reports, Hereford genl. infirmary, hip disease.. 408
Hospital reports, mercy hospital, typhoid fever ........ 366
Holmes, E. L. M.D., the audiphone and kindred instruments ........ 363
Hospital reports ........ 286
Hour-glass contraction previous to delivery of the child, by J. R. Moore, M.D ........ 253
Hotz, F. C., M.D., Wiederscheimer's methods of preserving anatomical preparations and whole bodies ........ 173
Hotz, F. C., M.D., a clinical lecture by, on the operation for inversion of the lower eyelid. 1
Hurd, Dr. H. H ........ 266
Hydrate of chloral and oxide of zinc in acute intestinal affections of children ........ 614
Hydrocele treated by an injection of the perchloride of iron. 520
Hydrobromic ether as an anæsthetic ........ 429

PAGE.
Hygiene and public health, edited by A. H. Buck, M.D ..... 89

I.

Ingals, E. F., M.D., cases of tracheotomy, for croup and diphtheria ........ 459
Ingals, E. F., M.D., the early treatment of burns and scalds. 249
Internes of Cook Co. hospital alumni association ........ 443
Intemperance, the cinchona cure for, by Chas. Warrington Earle, M.D ........ 128
Illinois state board of health, examinations of ........ 620
Illinois state medical society.. 446
Infirmary, Illinois charitable eye and ear, clinic at, by Prof. S. J. Jones ........ 113
Illinois State board of health.. 101
Iowa State university, medical department of ........ 441
Iron, salicylate of ........ 81
Intussusception probably, obstruction of bowel relieved by aspiration, by H. F. Godfrey, M.D ........ 373

J.

Japanese surgery and medicine, by Edmund Andrews, M.D... 87
Jones, S. J., Prof., notes from clinic at Ills. charitable eye and ear infirmary ........ 113

L.

Letter from Vienna, by W. T. Belfield, M.D ........ 619
Letter from Boston ........ 521
Lee, E W., M.D., on tetanus.... 516
Letter from Boston ........ 168
Lee, E. W., MD., and Chr. Fenger, M.D., select topics of modern surgery; illustrated by cases ........118 465
Lithotomy, report of a case of, by A. E. Baldwin, M.D ........ 73
Local sanitary conditions and epidemics ........ 534
Lyman, H. M., M.D., diseased meats and foul water considered as causes of disease.. 588
Lydston, G. Frank, M.D., the anomalous origin of the descendens noni ........ 263

M.

Marine hospital service ........ 222
Medicine, science and art of, by N. S. Davis, M.D., LL.D ........ 449

PAGE.
Mercury, chemistry and toxicology of, by S. V. Clevenger, M.D. ... 337
Medical charities ... 281
Meacher, Wm., M.D., the value of ergot in the treatment of pneumonia ... 260
Meacham, J. G., Jr., M.D., osteocephaloma of the thigh ... 205
Metric system, the ... 100
Microscopical society of Illinois ... 613
Michigan State board of health 609
Milk, on the assimilation of, in the new-born ... 111
Moore, J. R., M.D., hour-glass contraction *previous* to the delivery of the child ... 253

N.

National board of health, annual report of the ... 193
New-born, retention of life in the ... 619
New York letter ... 269
New-born, on the assimilation of milk in the ... 111
Newkirk, G., M.D., dental hints for country practitioners ... 63

O.

Obstetrics and gynæcology, the diagnosis of large ovarian tumors ... 554
Osteocephaloma of the thigh, by J. E. Meacham, Jr., M.D. . 205
Osteo-myeletis, spontaneous, of the long bones, by N. Senn, M.D. ... 10
Oxide of zinc and hydrate of chloral, in the acute intestinal affections of children ... 614
Obituary, Surg. Gen. E. B. Wolcott ... 215
Otology and ophthalmology ... 663, 665, 666, 667, 668, 669, 670

P.

Practical medicine:
Treatment of uræmia by injections of pilocarpin ... 654
Practical medicine:
Cure of naso-pharyngeal polypi by interstitial injections of chloride of zinc ... 653
Observations on the digestion of milk ... 652
Experimental study of the treatment of hepatic colic .. 651
Precocity ... 651

PAGE.
Local morbid temperatures in affections of the abdomen .. 646
A simple method of cure for ozœna ... 646
Deformities of the vulva produced by defloration, masturbation, saphism and prostitution ... 642
Phagedenic chancre, pyrogallic acid in ... 641
Lecture on epilepsy, by W. R. Gowers, M.D., F.R.C.P ... 550
Practice, notes from private, D. A. K. Steele, M.D ... 158
Pratt, G. B., M.D., my struggle with a tape-worm, by ... 77
Perinephritis, in children, fifteen cases, making a total of twenty-eight cases, by V. P. Gibney, M.D ... 561
Perchloride of iron, hydrocele treated by an injection of ... 520
Pellizari, Dr. Celso, on epileptiform syphilis ... 492
Pneumonia, blood-letting in the treatment of, by T. F. Worrell, M.D ... 354
Pneumonia, the value of ergot in the treatment of, by William Meacher, M.D ... 260
Pilcher, L. S., M.D., double monsters ... 416
Phthisis, is it self-limiting? by William Porter, A.M., M.D ... 103
Prolapsus of the rectum, treatment of, by hypodermic injections of ergotine ... 599
Poison by chloras potassii, by Dr. A. Barlow ... 518
Pott's disease, the mechanical treatment of, by Dr. George F. Somers ... 256
Porter, William, A.M., M.D., is phthisis self-limiting? ... 103
Puerperal eclampsia, a severe case of, at the eighth month of pregnancy, recovery, by E. Day, M.D ... 597
Pyrogallic acid in phagedenic chancre ... 641
Philadelphia letter ... 82
Physiology of the plastic art, a contribution to, by Dr. James I. Tucker ... 175

R.

Reports of societies, Brainard district medical society, J. A. Walker, secretary ... 376

PAGE.
Reeve, J. C., M.D., two new anæsthetics.................... 628
Rush medical college, commencement exercises of...... 435
Rush medical college, concours at.......................... 117

S.

Salicylic acid in treatment of rheumatic diseases of the eye, by F. C. Hotz, M.D........... 501
Smart, Charles, M.D., investigations concerning water supply at Memphis, and other places in Tennessee and Mississippi, under direction of the National board of health....... 394
Small-pox, a suggestion, by John Conant........ ............ 274
Salicylate of iron............. 81
Seiffert, Dr. Rudolph, the treatment of diphtheria.......... 166
Steele, Dr. D. A. K., notes from private practice............. 158
Senn, N., M.D., spontaneous osteo-myelitis of the long bones...................... 10
Spina bifida tumor, excision of, cure, by W. H. Fitch, M.D.... 371
Society reports:
Illinois State medical society. 600
Transactions of the Chicago gyneæcological society..... 509
Somers, Dr. George F., the mechanical treatment of Pott's disease.................. 256
Surgery:
The Porro operation. .. .... 656
Ablation of a goitre; cure in six days.............. ... 654
Select topics of modern, by Drs. E. W. Lee and Chr. Fenger............... .118 465
Summary:
Practical medicine, surgery and therapeutics.......... 430
Physiology, practical medicine and therapeutics...... 318
Syphilis, epileptiform, by Dr. Celso Pellizari.............. 492

T.

Tracheotomy, cases of, for croup and diphtheria, by E. F. Ingals, M.D............................. 594
Tape-Worm:
Pumpkin seed for, by H. J. Thomas, M.D.............. 173
My stru gle with a, by G. B. Pratt, M.D ................ 77

PAGE.
Therapeutics:
The topical uses of ergot.... 670
Tannate of pelletierin as a tæniafuge................. 558
Rhus toxicohendron......... 558
Hydrofluoric inhalations in diphtheria................. 558
Tetanus, E. W. Lee, M.D., on.... 516
Thermometer, a new surface, by D. R. Brower, M.D........... 505
Therapeutics, mechanical chemistry and toxicology of mercury, by S. V. Clevenger, M.D. 337
Tetanus, traumatic, by Henry Van Buren, M.D.......... ... 163
Triplets, a case of, by J. B. Crandall, M.D.................... 507
Thomas, H. J., M.D., was it dilatation of the left ventricle.... 374
Thomas, H. J., M.D., pumpkin seed for tape worm .......... 173
Tucker, Dr. James I........... 383
Tucker, Dr. James I., a contribution to the physiology of the plastic art.... ............... 175
Tucker, Dr. James I., gynæcology in the 17th century....... 154

U.

Urethra, follicular abscess of, by Fessenden, N. Otis, M.D...... 459

V.

Vaughan, Victor C., M.D., Ph. D., the contamination of drinking water by filtration of organic matter through the soil...... 635
Van Buren, Henry, M.D., traumatic tetanus .......... ..... 163
Ventricle, was it dilatation of the left? by H. J. Thomas, M.D.... 374
Vienna letter, by W. T. Bellfield, M.D.... ............... 619

W.

Water Supply, of Memphis and other places, by Charles Smart, M.D.................... ........ 394
Water, on the use of, in the treatment of diseases of the skin, by L. Duncan Bulkley, M.D.. 52
Wolcott, Surg. Gen'l., obituary. 215
West Chicago medical society:
E. W. Lee, M.D., on tetanus... 516
White, J. C., M.D., a fourth year in the medical school........ 547
Woman's Med. college, Chicago 442
Worrell, T. F., M.D., blood-letting in the treatment of pneumonia 435

### BOOKS AND PAMPHLETS RECEIVED.

Homœopathy: What is It? By A. B. Palmer, M.D. Detroit: Geo. S. Davis. 1880.

Foreign Bodies in Surgical Practice. By A. Poulet, M.D. Two volumes. New York: Wm. Wood & Co. 1880.

Venereal Diseases, Including Stricture of Male Urethra. By E. L. Keyes, M. D. New York: Wm. Wood & Co. 1880.

Physical Diagnosis (Hand Book). By Dr. Paul Guttmann. New York: Wm. Wood & Co. 1880.

Atlas of Histology. By E. Klein, M.D., and E. Noble Smith, M.D. Part X. Philadelphia: J. B. Lippincott & Co. 1879.

Ethylization: The Anæsthetic Use of the Bromide of Ethyl. By R. J. Lewis, M. D. (Reprint from Medical Record.)

Modern Abuse of Gynæcology. By Clifton E. Wing, M.D., Boston.

Rectal Feeding in Disease. By W. W. Potter, M.D., Batavia, N. Y.

Thirteenth Annual Report of Cincinnati Board of Health, for the year ending Dec. 3, 1879.

Importance of Anal Fissure in the Treatment of Diseases of Women. By R. S. Sutton, M.D., Chicago.

Transactions of American Dental Association, Nineteenth Annual Session Chicago: Knight & Leonard.

A Case of Intrauterine Ichthyosis. By Wm. R. Smith, Sr., M.D., Cairo, Ills. Three wood cuts.

---

THE late concours held at Rush Medical College for the position of Clinical Adjunct to the Chair of Diseases of Children, resulted in the appointment of Dr. J. Suydam Knox, of Chicago, who is already well known to the profession as a scientific man, and, moreover, highly esteemed socially.

### ANNOUNCEMENTS FOR THE MONTH.

### SOCIETY MEETINGS.

Chicago Medical Society—Mondays, June 7 and 21.

West Chicago Medical Society—Mondays, June 14 and 28.

### CLINICS.

MONDAY.

Eye and Ear Infirmary—2 p. m., Ophthalmological, by Prof. Holmes; 3 p. m., Otological, by Prof. Jones.

Mercy Hospital—2 p. m., Surgical, by Prof. Andrews.

Rush Medical College—2 p. m., Dermatological and Venereal, by Prof. Hyde; 3 p. m., Medical, by Dr. Bridge.

Woman's Medical College—2 p. m., Dermatological and Venereal, by Prof. Maynard; 3 p. m., Diseases of the Chest, Prof. Ingals.

TUESDAY.

Cook County Hospital—2 to 4 p. m., Medical and Surgical Clinics.

Mercy Hospital—2 p. m., Medical, by Prof. Quine.

WEDNESDAY.

Chicago Medical College—2 p. m., Eye and Ear, by Prof. Jones.

Rush Medical College—3:30 to 4:30 p. m., Diseases of the Chest, by Dr. E. Fletcher Ingals.

THURSDAY.

Chicago Medical College—2 p. m., Gynæcological, by Prof. Jenks.

Rush Medical College—3 p. m., Diseases of the Nervous System, by Prof. Lyman.

Eye and Ear Infirmary—2 p. m., Ophthalmological, by Dr. Hotz.

Woman's Medical College—3 p. m., Surgical, by Prof. Owens.

FRIDAY.

Cook County Hospital—2 to 4 p. m., Medical and Surgical Clinics.

Mercy Hospital—2 p. m., Medical, by Prof. Davis.

SATURDAY.

Rush Medical College—2 p. m., Surgical, by Prof. Gunn.

Chicago Medical College—2 p. m., Surgical, by Prof. Isham; 3 p. m., Neurological, by Prof. Jewell.

Woman's Medical College—11 a. m., Ophthalmological, by Prof. Montgomery; 2 p. m., Gynæcological, by Prof. Fitch.

Daily Clinics, from 2 to 4 p. m., at the Central Free Dispensary, and at the South Side Dispensary.

